# Oxytocin facilitates valence-dependent valuation of social evaluation of the self

**DOI:** 10.1038/s42003-020-01168-w

**Published:** 2020-08-13

**Authors:** Danyang Wang, Yina Ma

**Affiliations:** State Key Laboratory of Cognitive Neuroscience and Learning, IDG/McGovern Institute for Brain Research, Beijing Key Laboratory of Brain Imaging and Connectomics, Beijing Normal University, Beijing, 100875 China

**Keywords:** Human behaviour, Social neuroscience

## Abstract

People are eager to know the self in other’s eyes even with personal costs. However, what drives people costly to know evaluations remains unknown. Here we tested the hypothesis of placing subjective value on knowing social evaluations. To quantify the subjective value, we developed a *pay-to-know* choice task where individuals trade off profits against knowing social evaluations. Individuals computed independent unknown aversion towards positive and negative social evaluations and placed higher values on knowing social evaluation on positive than negative aspects. Such a valence-dependent valuation of social evaluation was facilitated by oxytocin, a neuropeptide linked to feedback learning and valuation processes, by decreasing values of negative social evaluation. Moreover, individuals scoring high in depression undervalued positive social evaluation, which was normalized by oxytocin. We reveal the psychological and computational processes underlying self-image formation/update and suggest a role of oxytocin in normalizing hypo-valuation of positive social evaluation in depression.

## Abstract (web summary)

Danyang Wang and Yina Ma measure the amount of money participants are willing to forgo for the opportunity to access social or non-social evaluations of the self. They show that subjective values on knowing social evaluation is valence-dependent whereas that on non-social evaluation is valence-insensitive. Moreover, oxytocin contributes to valence-dependent valuation on social evaluation.

## Introduction

Humans are curious about how the self is evaluated—either praised or criticized. Individuals spend over 70% of their daily conservation expressing their opinions about others and getting to know the self through the eyes of their friend, colleague, and employer^1–4^. We also frequently check social networking sites (such as Facebook and Twitter) to see how many people ‘like’ our pages or how others comment on our recent posts. However, it remains unknown what drives people to wonder how the self is evaluated, even at a personal cost. In the current study, we propose that the subjective value individuals place on the opportunity to know evaluations of the self drives costly-to-know behavior. Three lines of research motivate this hypothesis. First, feedback on the self (e.g., praise and criticism) provides guidance for how to behave and/or interact appropriately with others^1,2^ and helps with individuals’ reputation formation and management^5,6^. Second, people do not tolerate ambiguity in self-related information; thus, the curiosity (of perceived self-image) provides an internal, psychological force driving individuals to explore how the self is evaluated^2,7,8^. Third, evaluative information conveys motivational value and has been used to motive expectation, learning, and decision-making^9,10^. Taken together, we hypothesized that subjective value would be assigned to knowing evaluation.

To test this hypothesis, we develop a pay-to-know choice task where participants make a series of choices between two alternative options: ‘to-know’ (TK) and ‘not-to-know’ (NTK) the evaluations. The two options differed in the amount of monetary reward received. The pay-to-know choice task is modified from the “pay-per-view” paradigm. Using this paradigm, previous work has shown that viewing social signals or self-disclosure is intrinsically rewarding in that monkeys would sacrifice juice for the opportunity to view another monkey;^11,12^ humans forgo monetary rewards to view attractive individuals of the opposite sex^13^ or to disclose information about oneself^14^. The current pay-to-know choice task allows us to measure the amount of money participants are willing to forgo to the opportunity to access evaluations from other people or a computer program and allows us to quantify the subjective value individuals assigned to knowing the evaluations. Moreover, integrating computational modeling, we sought to dissect the contribution of monetary reward and the opportunity to know evaluation in making choices in the pay-to-know task. Individual’s subjective cost of not-knowing was characterized by a subject-specific unknown aversion parameter derived from a computational model of the individual’s choices.

Next, we asked the question of whether the subjective value assigned to the opportunity to know evaluation would be modulated by the valence of the evaluation aspects. The evaluation of the self functions to correct and guide one’s behavior^2,15^ and shapes one’s self-image^16^. Positive feedback reinforces the right behavior or choice, and negative feedback helps to avoid future error or failure; thus, both positive and negative evaluations of the self could be valuable. This could be especially true for evaluations based on objective standard criteria^17^. However, feedback has other functions besides telling right from wrong, especially social feedback. Social feedback (such as evaluation from other people) conveys different social signals depending on the valence. For example, positive social evaluation provides social support and social approval and facilitates social connection^18,19^ whereas negative social evaluation signals social threat and can hurt one’s feelings^20,21^. Thus, we hypothesize that the value of knowing nonsocial evaluation would be insensitive to valence, whereas people would be more motivated to know positive rather than negative social evaluations (the valence-dependent valuation of social evaluation was preregistered via Open Science Framework, OSF, https://osf.io/ezxws).

Finally, we aim to reveal the molecular substrates modulating the subjective value placed on evaluation. Oxytocin is of particular interest in the current study as oxytocin has been linked to social feedback learning and valuation processes^22,23^. Specifically, intranasal administration of oxytocin increased the learning of positive social feedback and decreased the learning of negative feedback. Moreover, animal work has shown that oxytocin decreased the value placed on the opportunity to view socially threatening stimuli (i.e., faces of dominant monkeys)^24^. These findings lead us to predict that individuals given oxytocin would increase the value assigned to positive social feedback and especially reduce the value of negative social feedback, which was preregistered via OSF. Moreover, we examined individual differences in oxytocin effect on the valuation of social feedback. It has been suggested that oxytocin effect is sensitive to individual differences^23,25,26^, with a stronger oxytocin effect on less socially adapted individuals^26^, such as individuals who scored high in depression or anxiety^22,27^. It has also been shown that depressive individuals are characterized by a negative bias^28,29^ and reduced learning of positive social feedback^30^. Thus, the current study further examined whether the oxytocin effect on the valuation of social feedback was modulated by individuals’ depressive scores.

By measuring the amount of money that individuals would forgo for the opportunity to know social or nonsocial evaluations, we tested the hypothesis that the subjective value placed on evaluation drives the costly-to-know behavior. The results from five experiments lent cognitive and computational support for our hypothesis. Furthermore, the subjective value was modulated by the source and valence of the evaluation. Specifically, participants equally valued positive and negative nonsocial evaluations, characterized by a shared unknown aversion computation. However, individuals computed independent unknown aversion towards positive and negative social evaluations and placed a higher value on the opportunity to know another person’s evaluation on positive than negative aspects. Moreover, such a valence-dependent valuation of the social evaluation was facilitated by oxytocin.

## Results

### Paying for the opportunity to know social evaluation

**Fig. 1:**
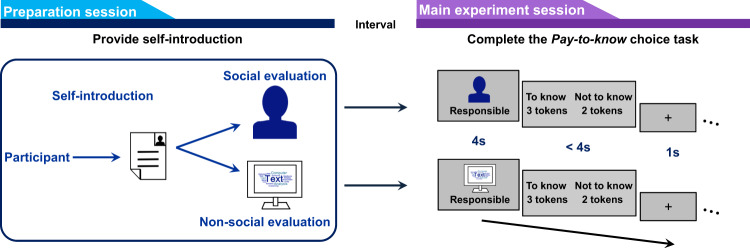
Illustration of the general experimental procedure. Participants were first invited to a preparation session to complete self-introduction as materials for social (i.e., other people) and nonsocial (i.e., a computer program) evaluations. About 4 days later (mean interval across participants: mean ± SD = 3.86 ± 4.42 days), participants came to the main experiment session and completed the pay-to-know choice task. For each trial of the pay-to-know task, participants were first presented with a single aspect (a positive or negative trait word) on which he could receive an evaluation and then had to decide whether they wanted to know the evaluation of the presented aspect or not. The ‘to-know’ (TK) and ‘not-to-know’ (NTK) options were associated with different monetary rewards (payoff difference ranged from −3 to 3).

Participants were invited to trade off different amounts of monetary reward (Supplementary Table 1 for the payoff matrix) against the opportunity to have access to evaluations either provided by other people or a computer program (i.e., the pay-to-know choice task, Fig. 1). We first examined the extent to which participants would pay for the opportunity to know social evaluation (Exp. 1, *n* = 36 males). If knowing social evaluation is intrinsically motivating, we would expect participants to forgo monetary reward to choose the ‘to-know’ option; otherwise, participants would consistently choose whichever option was associated with higher monetary rewards to maximize their financial payoff. We found that participants generally chose to know social evaluations in more than 50% of the trials (63.57% ± 13.47% vs. 50%, *t*(35) = 6.05, *p* = 6.74 × 10^−7^, 95% confidence interval (CI), 0.09 to 0.18, Cohen’s *d* = 1.01). Next, we focused on the trials where choosing the ‘to-know’ option gains no more money than choosing the ‘not-to-know’ option but was associated with monetary cost (i.e., *M*_*TK*_ ≤ *M*_*NTK*_, costly-to-know trials). Participants chose costly to know social evaluations 43.3 ± 2.55% of the time (referred to as the costly knowing ratio).

**Fig. 2:**
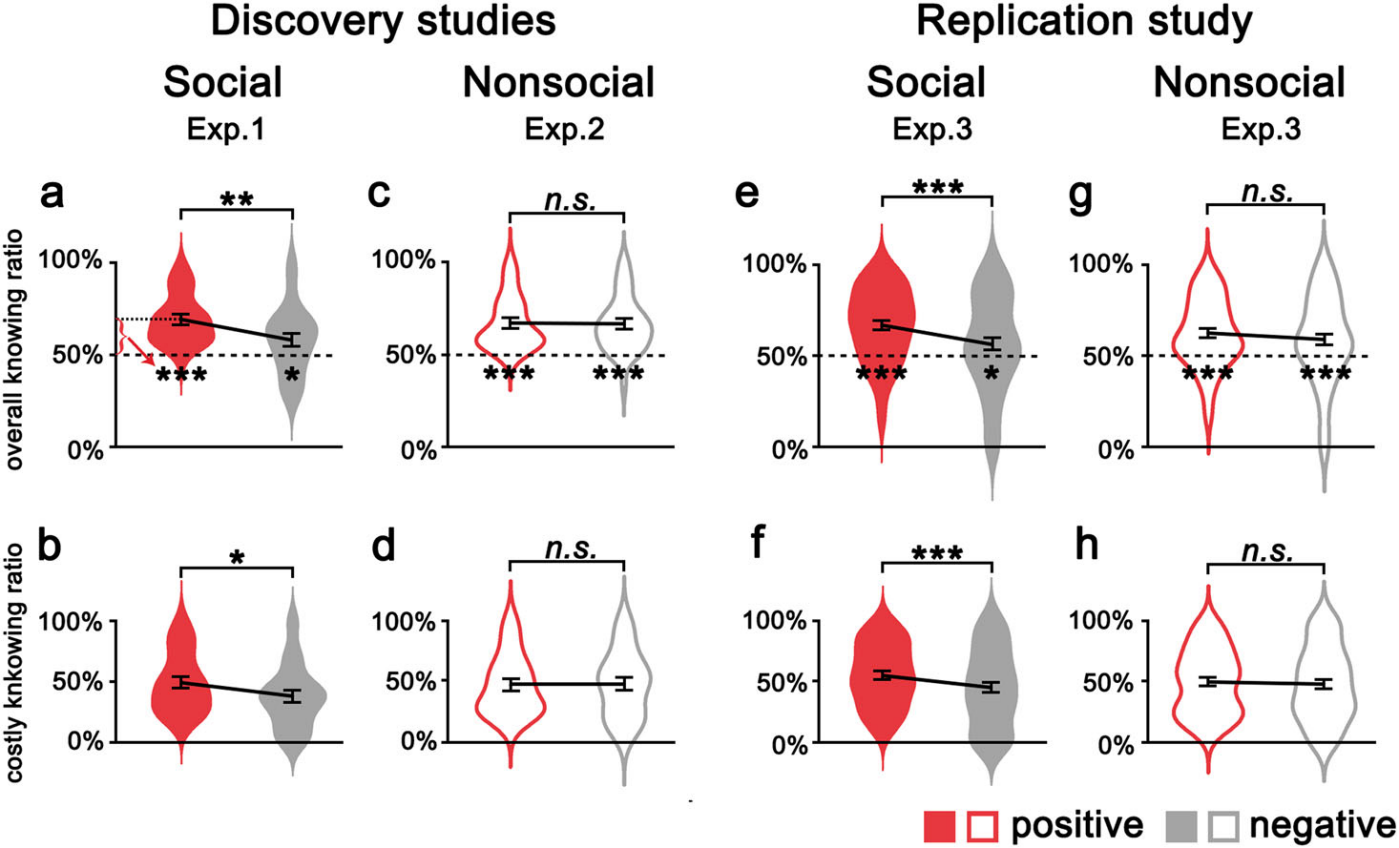
The model-free results in Exp. 1–3. Participants preferred to pay more to know social evaluations of positive than negative aspects (**a**, **b***, n=* 36 participants) whereas they would forgo a similar amount of money to the opportunity to know positive and negative nonsocial evaluations (**c, d***, n=* 36 participants). These results were replicated in online Exp. 3 (**e**–**h**, *n* = 98 participants for the social condition, *n* = 110 participants for the nonsocial condition). The violin plots indicate the distribution of indices from the pay-to-know choice task, with elements inside the violin plots representing the mean and standard error. (*p < 0.05, **p < 0.01, and ***p < 0.001; n.s. not significant).

We then examined whether participants would equally prefer to know social evaluation on positive and negative aspects. If so, we would expect participants to forgo similar amounts of monetary reward to the opportunity to know either positive or negative aspects. We found that while participants valued the opportunity to know evaluations on both positive (69.15 ± 13.77% vs. 50%, *t*(35) = 8.35, *p* = 7.63 × 10^−10^, 95% CI, 0.15–0.24, Cohen’s *d* = 1.39) and negative (57.99 ± 19.11% vs. 50%, *t*(35) = 2.51, *p* = 0.017, 95% CI, 0.02–0.14, Cohen’s *d* = 0.42) aspects, participants preferred to know positive social evaluations more than negative ones (*t*(35) = 3.42, *p* = 0.002, 95% CI, 0.05–0.18, Cohen’s *d* = 0.57, Fig. 2a). Moreover, participants more often chose costly-to-know positive (vs. negative) evaluations (higher costly knowing ratio: 49.01 ± 24.68% vs. 37.65 ± 26.42%, *t*(35) = 2.83, *p* = 0.008, 95% CI, 0.03–0.20, Cohen’s *d* = 0.47, Fig. 2b).

### Paying to know social vs. nonsocial evaluations

Next, we asked whether participants placed subjective value on knowing social evaluation on themselves or on knowing any opinions of themselves. We conducted Exp. 2 with an independent sample (*n* = 36 males), following the same procedure as Exp. 1 except that participants were told that the evaluations were the outputs from the text/facial analysis of their self-introduction materials. We found that participants chose the ‘to-know’ option in 66.80 ± 13.49% of the trials (vs. 50%, *t*(35) = 7.48, *p* = 9.39 × 10^−9^, 95% CI, 0.12 to 0.21, Cohen’s *d* = 1.25). For the costly-to-know trials, participants chose costly ‘to-know’ 46.91 ± 24.21% of the time. Moreover, participants preferred to know nonsocial evaluations on both positive and negative aspects (66.97 ± 14.08% vs. 66.63 ± 15.16%, *t*(35) = 0.18, *p* = 0.859, 95% CI, −0.04 to 0.04, Cohen’s *d* = 0.03, Fig. 2c). The cost of knowing evaluations on positive and negative aspects was comparable (costly knowing ratio: 46.48 ± 24.69% vs. 47.35 ± 27.39%, *t*(35) = −0.27, *p* = 0.790, 95% CI, −0.07 to 0.06, Cohen’s *d* = −0.04, Fig. 2d).

These results suggested that while individuals preferred to know both social and nonsocial evaluations, wanting to know social evaluation is valence-dependent whereas wanting to know nonsocial evaluation is valence-insensitive. To further confirm this difference, we conducted ANOVA on knowing ratio, with valence (positive vs. negative) as a within-subjects factor and the evaluation source (social vs. nonsocial) as a between-subject factor. This analysis showed a significant main effect of valence (*F*(1,70) = 9.30, *p* = 0.003, *η*^*2*^ = 0.12) and a significant valence-by-source interaction (*F*(1,70) = 8.24, *p* = 0.005, *η*^*2*^ = 0.11), confirming that the valence-dependent value was selective for social (but not nonsocial) evaluations. Moreover, the interaction between evaluation source and valence remained reliable after controlling for desirability rating (three participants from Exp. 2 did not complete the desirability rating, leaving 69 participants in this analysis), positive and negative affect, between-session interval, valence rating, and self-relevance rating (*F*(1,61) = 5.76, *p* = 0.019, *η*^*2*^ = 0.09).

To specifically examine whether the willingness to know positive or negative evaluations was influenced by the evaluation source, we conducted simple effect analyses on the knowing ratio. The knowing ratio for evaluation of negative aspects was lower for social sources than for nonsocial sources (*F*(1,70) = 4.52, *p* = 0.037, *η*^*2*^ = 0.06), whereas the knowing ratio for evaluation of positive aspects was not influenced by evaluation source (*F*(1,70) = 0.44, *p* = 0.508). Similarly, valence (positive vs. negative) × evaluation source (social vs. nonsocial) ANOVA on costly knowing behavior also revealed a significant valence main effect (*F*(1,70) = 4.14, *p* = 0.046, *η*^*2*^ = 0.01) and a valence-by-source interaction (*F*(1,70) = 5.63, *p* = 0.020, *η*^*2*^ = 0.07), as the effect of valence on costly knowing behavior was conditioned by evaluation source. In addition, we asked participants to report the valence and self-relevance of each trait word, and there was no difference in the rating scores between the social and nonsocial evaluations (Exp. 1 vs. 2: *ps* > 0.10).

### Independent replication

We performed an independent online experiment with a large sample (Exp. 3, *n* = 208 males), aiming to provide replication. Moreover, we additionally included a situation where both the ‘to-know’ and ‘not-to-know’ options were associated with monetary loss to test whether participants were also willing to lose more money for knowing evaluation. We provided a replication of our findings that individuals indeed prefer costly ‘to-know’ social evaluations (in a valence-dependent way, to a greater degree for positive than negative aspects, Fig. 2e, f) and nonsocial evaluations (regardless of valence, equally for positive and negative aspects, Fig. 2g, h), both in the monetary gain and loss situations (Supplementary Fig. 1). Given that no difference was found between the monetary gain and loss situations, we thus narrowed further analyses to monetary gain situation, and only included the monetary gain situation in Exp. 4 and 5.

### Quantifying the subjective value of evaluation

**Fig. 3:**
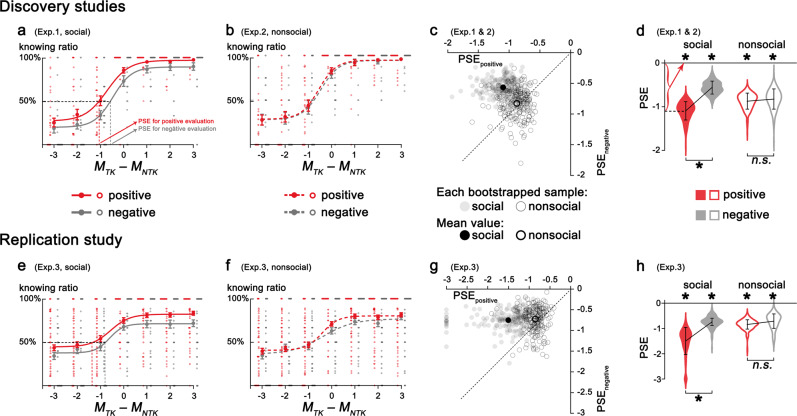
Subjective value assigned to social and nonsocial evaluations. The proportion of choosing to know the social (**a**, *n=* 36 participants; **e**, *n=* 98 participants) or nonsocial (**b**, *n=* 36 participants; **f**, *n=* 110 participants) evaluations was plotted against the monetary difference in the ‘to-know’ and ‘not-to-know’ options and fitted with sigmoid functions for the discovery and replication experiments, respectively (error bars represent standard errors across participants in each condition; each circle represents the knowing ratio for each participant in each condition). The PSE between ‘to-know’ and ‘not-to-know’ options was calculated. **c** (discovery sample, 200 bootstrapped samples) and **g** (replication sample, 200 bootstrapped samples) show the distributions of 200 bootstrapped sample means for social and nonsocial evaluations plotted against the PSE for evaluations of positive and negative aspects. Each gray dot represents the mean value of a bootstrapped sample, and dashed lines indicate the diagonal (the black dot indicates the mean value of 200 bootstrapped samples). Points on the diagonal represent bootstrapped samples with equal subjective value for knowing evaluations of positive and negative aspects. Points above the diagonal indicate bootstrapped samples with higher value assigned to knowing evaluations of positive than negative aspects, and points below represent samples assigning higher value to knowing evaluations of negative than positive aspects. The PSE of social evaluation was significantly higher for positive than for negative aspects, whereas the PSEs of nonsocial evaluation of positive and negative aspects were comparable (**d** for discovery sample, *n=* 72 participants; and **h** for the replication sample*, n=* 208 participants). The violin plots indicate the distribution of the PSEs, with elements inside representing the mean and bootstrap standard error (asterisk and *n.s*. represent significant/insignificant in a bootstrap test).

We next quantified the subjective value individuals placed on the opportunity to receive social and nonsocial evaluations by calculating the point of subjective equivalence (PSE) where participants were equally likely to choose the ‘to-know’ and ‘not-to-know’ options. We plotted the proportion of ‘to-know’ choices against the relative payoff for the ‘to-know’ than ‘not-to-know’ options (i.e., Δ*M* = *M*_*TK*_ – *M*_*NTK*_, Fig. 3a, b) and fitted participants’ choice data with sigmoid functions through a bootstrap procedure with 200 iterations similar to previous study^31^. The *x*-axis represents payoff differences between the two options (i.e., Δ*M*: −3, −2, −1, 0, 1, 2, 3), *y*-axis indicates the percentage of trials choosing to know at each payoff difference situation. The PSE was calculated as the point on the abscissa where the sigmoid fitted curve passed 50% on the ordinate, indicating the amount of tokens gained or lost when participants was equally likely to choose ‘to-know’ and ‘not-to-know’. Negative PSE values indicate that participants incurred a relative monetary loss for the opportunity to know, i.e., ‘to-know’ was worth more tokens than ‘not-to-know’.

PSEs were derived from fitting sigmoid functions to each participant’s choices, representing the relative subjective value of ‘to-know’ over ‘not-to-know’ social (Fig. 3a) or nonsocial (Fig. 3b) evaluations. PSEs were significantly smaller than zero, suggesting that knowing social and nonsocial evaluations was worth significantly more than not-knowing (Fig. 3c). The subjective value assigned to the opportunity to know evaluation equals earning 0.791 tokens on average for social evaluation (bootstrap 95% CI, −1.037 to −0.539) and 0.837 tokens for nonsocial evaluation (bootstrap 95% CI, −1.168 to −0.435). Moreover, the subjective value of social evaluation on positive aspects (PSE_positive_ = −1.057) was significantly higher than that for negative aspects (PSE_negative_ = −0.571, PSE_positive *vs*. negative_: bootstrap 95% CI, −0.861 to −0.101, Fig. 3c, d). However, the subjective values assigned to nonsocial evaluation for positive (PSE_positive_ = −0.838) and negative (PSE_negative_ = −0.811) aspects were comparable (PSE_positive *vs*. negative_: bootstrap 95% CI, −0.303 to 0.264, Fig. 3c, d). The same pattern was replicated in Exp. 3 (social: PSE_positive_ = −1.506 vs. PSE_negative_ = −0.749, bootstrap 95% CI, −0.966 to −0.114; nonsocial: PSE_positive_ = −0.809 vs. PSE_negative_ = −0.797, bootstrap 95% CI, −0.513 to 0.408; Fig. 3e–h).

### Computations underlying the valuation of evaluation

To reveal the computations underlying the costly-to-know behavior, we built a range of computational models to fit participants’ choices (Supplementary Methods), such as modified temporal-discounting models (which considered a temporal discount rate for the value of evaluation^32–34^), and models that estimated loss aversion for money. A softmax function was used to transform the trial-by-trial value into choice probability. We found that participants’ choices of knowing social or nonsocial evaluations were characterized by different computations.

Participants’ choices of knowing social evaluation or not were most parsimoniously explained by a model (Model 1) featuring distinct valuation of social evaluations on positive and negative aspects (i.e., *β*_*positive*_, *β*_*negative*_), together with a parameter (i.e., *α*) capturing the contribution of monetary payoff for the action choice:

$$\Delta V = \alpha {\mathrm{\Delta }}m + \beta {\mathrm{\Delta }}e$$

$$\beta = \left\{ {\begin{array}{*{20}{c}} {\beta _{{\mathrm{positive}}}\;{\mathrm{evaluation}}\;{\mathrm{on}}\;{\mathrm{positive}}\;{\mathrm{aspect}}} \\ {\beta _{{\mathrm{negative}}}\;{\mathrm{evaluation}}\;{\mathrm{on}}\;{\mathrm{negative}}\;{\mathrm{aspect}}} \end{array}} \right.$$

where Δ*V* is the subjective value difference between choosing the left and right options, Δ*m* and Δ*e* are the differences in monetary payoff (Δ*m* = *M*_*left*_ – *M*_*right*_) and to-know the evaluation or not between the two options (Δ*e* = 1, if left choice is ‘to know’; Δ*e* = −1, if left choice is ‘not to know’). *α* captures the subjective value of monetary payoff differences, and *β*_*positive*_ and *β*_*negative*_ are unknown aversion parameters that capture the subjective cost of not-knowing evaluation. It has been shown that while some individuals prefer to know the evaluations and avoid not-knowing, others may show an aversion towards knowing evaluations, both for potentially positive^35^ and negative evaluations^36^. Thus, we set *β* parameters ranging from −1 to 1. Positive values of *β* indicated that participants were averse to not-knowing (i.e., unknown aversion, *β* = 1 represents maximal aversion for unknown, maximizing the value of the ‘to-know’ option), whereas negative values of *β* suggested that participants were unlikely to know the evaluation (*β=* −1 represents maximal aversion for knowing, minimizing the value of the ‘to-know’ option).

**Fig. 4:**
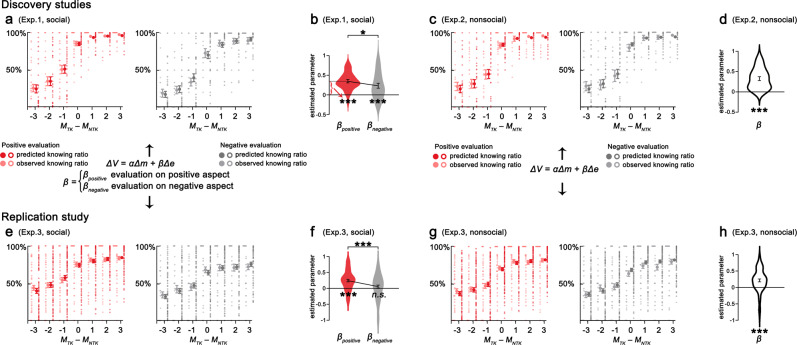
Computations underlying costly-to-know behavior. **a, b** Individuals’ choices in the pay-to-know social evaluation task were most parsimoniously explained by a model featuring the contribution of monetary payoff difference (*α*) and distinct contribution of knowing positive and negative social evaluations (*β*_*positive*_ > *β*_*negative*_) on the action choice (*n=* 36 participants). **c, d** In the nonsocial evaluation version, participants’ choices were best fitted by a model featuring the valuation of monetary payoff difference (*α*) and unknown aversion towards nonsocial evaluation (*β* > 0) (*n=* 36 participants). Observed data, light circles; predicted choice, dark circles (error bars represent standard errors across participants in each condition; empty circles represent value for each participant in each condition and solid circles represent mean value across participants in each condition). The winning model and the related results were replicated in the independent replication experiment (**e**–**h**, *n* = 98 participants for the social condition, *n* = 110 participants for the nonsocial condition). The violin plots indicate the distribution of the estimated parameters, with elements inside representing the mean and standard error. (*p < 0.05, ***p < 0.001, and n.s, not significant).

Model 1 outperformed a series of alternative models (Supplementary Table 2) and correctly predicted 88.25% of participants’ choices (95% CI, 85.64 to 90.86, Fig. 4a). The parameters *β*_*positive*_ and *β*_*negative*_ captured the impact of the desirability of knowing evaluations of positive and negative aspects on participants’ choices, respectively. The distinct valuation of knowing social evaluations of positive and negative aspects explained the model-free finding that participants traded different amounts of money to know social evaluation of positive and negative aspects.

The *β* parameter estimates derived from Model 1 were significantly above 0 (*β*_*positive*_ = 0.35, *t*(35) = 9.57, *p* = 2.61 × 10^−11^, 95% CI, 0.27–0.42, Cohen’s *d* = 1.60; *β*_*negative*_ = 0.23, *t*(35) = 4.17, *p* = 1.93 × 10^−4^, 95% CI, 0.12–0.34, Cohen’s *d* = 0.69, Fig. 4b), suggesting unknown aversion for social evaluations separately on positive and negative aspects. Moreover, *β* parameter estimates were higher for social evaluation of positive than negative aspects (*t*(35) = 2.31, *p=* 0.027, 95% CI, 0.01–0.22, Cohen’s *d* = 0.38, Fig. 4b). In addition, significantly more participants showed unknown aversion (i.e., *β* > 0) for social evaluations on positive (*N* = 35 out of 36) rather than negative (*N* = 26 out of 36) aspects (*χ*^2^ = 6.87, *p* = 0.009). These results suggested stronger aversion towards not-knowing positive (vs. negative) social evaluations.

Participants’ responses in choosing whether to know the nonsocial evaluation or not were parsimoniously explained by Model 2 (outperformed Model 1 and other models, Supplementary Table 2), with two parameters accounting for the value of to-know nonsocial evaluation (*β*) and monetary reward (*α*): Δ*V* = *α*Δ*m* + *β*Δ*e*. In this model, *β* is the unknown aversion parameter capturing the subjective cost of not-knowing evaluation, shared by nonsocial evaluations on the positive and negative aspects. The winning Model 2 correctly predicted 87.75% of participants’ choices on whether to know nonsocial evaluation (95% CI, 84.04 to 91.45%, Fig. 4c). The *β* parameter estimate derived from Model 2 was significantly above 0 (*β* = 0.32, *t*(35) = 7.81, *p* = 3.53 × 10^−9^, 95% CI, 0.24 to 0.41, Cohen’s *d* = 1.30, Fig. 4d), suggesting unknown aversion for nonsocial evaluation. The shared unknown aversion for positive and negative aspects provided a computational explanation for the valence-insensitive subjective value of nonsocial evaluation. In addition, the winning model and the pattern of the parameter estimates derived from the winning model for both social and nonsocial evaluations were replicated in the independent online experiment (Exp. 3, *n* = 208, Fig. 4e–h, Supplementary Table 2).

### Oxytocin effect on the valuation of social evaluation

**Fig. 5:**
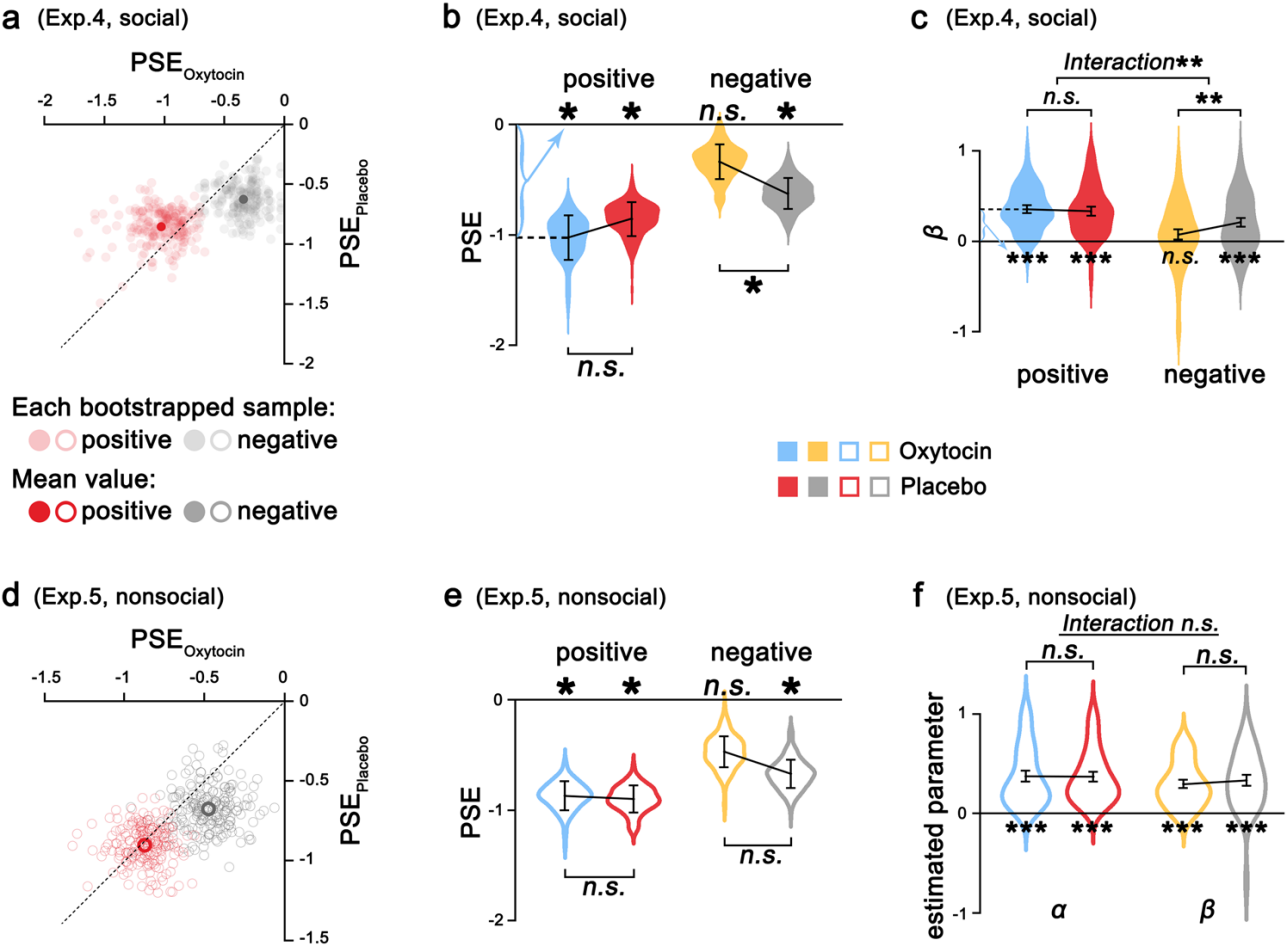
Oxytocin effect on the valuation of social evaluation. **a** (Exp. 4, social evaluation) and **d** (Exp. 5, nonsocial evaluation) show the distributions of 200 bootstrapped sample means for evaluations of positive and negative aspects plotted against the PSE in placebo and oxytocin treatment. **b** The effect of oxytocin on subjective value placed on positive and negative social evaluations (*n* = 56 participants). Oxytocin decreased the PSE for social evaluation on negative aspects but did not influence that of social evaluation on positive aspects. **c** Oxytocin decreased unknown aversion for social evaluation on negative aspects but not for social evaluation on positive aspects (*n* = 56 participants). **e, f** No significant effect of oxytocin was found for nonsocial evaluation. (*n* = 39 participants, *p < 0.05, **p < 0.01, and n.s. not significant). In **a** and **d**, each dot represents the mean value of a bootstrapped sample, and dashed lines indicate the diagonal. Points on the diagonal represent bootstrapped samples placing equal subjective value on knowing evaluation in the oxytocin and placebo conditions. Points above the diagonal indicate bootstrapped samples with higher value assigned to knowing evaluation under oxytocin than placebo treatment, and points below the diagonal represent samples assigning lower value to knowing evaluation under oxytocin than placebo treatment. The violin plots indicate the distribution of the PSE, with elements inside representing the mean and bootstrap standard error (*p < 0.05, **p < 0.005, and *n.s.* not significant).

We then conducted Exp. 4 (*n* = 56 males) to examine the modulation of oxytocin on the subjective value placed on knowing social evaluations. We first quantified the oxytocin effect on subjective values placed on positive and negative social evaluations by comparing PSEs under oxytocin and placebo. Oxytocin significantly decreased the subjective value of negative social evaluation (PSE_negative_, oxytocin vs. placebo: −0.326 vs. −0.625, bootstrap 95% CI, 0.033 to 0.622, Fig. 5a, b), even to the extent of having no subjective value assigned to negative social evaluation, as the PSE was not different from zero (PSE_negative-oxytocin_ = −0.339, bootstrap 95% CI, −0.626 to 0.040). However, the subjective value of positive social evaluation was not affected by oxytocin (PSE_positive_, oxytocin vs. placebo: −1.026 vs. −0.855, bootstrap 95% CI, −0.388 to 0.214, Fig. 5a, b).

Next, we examined the oxytocin effect on computational processes underlying the valuation of social evaluation. Participants’ choices regarding knowing social evaluation were also best fitted by Model 1. We found that oxytocin did not influence the weight of monetary payoff on participants’ choices (*α* parameter estimates derived from Model 1, oxytocin vs. placebo: *t*(55) = −0.89, *p=* 0.379, 95% CI, −0.07 to 0.03, Cohen’s *d* = −0.12). ANOVA on unknown aversion (*β)*, with treatment and valence as within-subjects factors, showed a significant interaction (*F*(1,55) = 8.11, *p* = 0.006, *η*^2^ = 0.13, Fig. 5c). While oxytocin did not significantly influence unknown aversion for positive social evaluation (*β*_*positive*_, oxytocin vs. placebo: *t*(55) = 0.62, *p=* 0.541, 95% CI, −0.05 to 0.10, Cohen’s *d* = 0.08), oxytocin significantly decreased unknown aversion for negative social evaluation (*β*_*negative*_, oxytocin vs. placebo: *t*(55) = −3.07, *p=* 0.003, 95% CI, −0.22 to −0.05, Cohen’s *d* = −0.41), even to the extent that participants given oxytocin did not show unknown aversion for negative social evaluation (*β*_*negative*_ under oxytocin was not significantly different from 0, *β*_*negative-oxytocin*_ = 0.07, *t*(55) = 1.45, *p* = 0.154, 95% CI, −0.03 to 0.17).

To further examine whether a similar effect of oxytocin would be observed for nonsocial evaluation, we conducted Exp. 5 (*n* = 36 males). Under placebo, we replicated the findings that participants similarly valued positive and negative nonsocial evaluations . Moreover, as predicted, no significant oxytocin effect was observed on the valuation of nonsocial evaluation (Fig. 5d–f).

We also showed that mood changes before and after the experiment (Supplementary Table 3, 4) and the decision times (Supplementary Table 5) were not different between the oxytocin and placebo sessions in both Exp. 4 and 5. In addition, we asked participants to report the valence and self-relevance rating of each trait word, and no difference in rating scores was found between the oxytocin and placebo sessions (*ps* > 0.05). These results suggested that the effect of oxytocin on the valuation of social evaluation cannot simply be attributed to the potential effects of oxytocin on mood, cognitive performance or ratings of the evaluation aspects.

### Oxytocin effect in individuals with high depressive scores

**Fig. 6:**
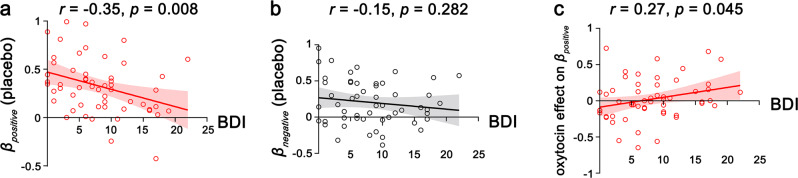
Individual differences in the valuation of social evaluation and the related oxytocin effect. **a**, **b** Under placebo, individuals scoring higher on the Beck Depression Inventory (BDI) showed less unknown aversion for positive **a** but not negative **b** social evaluations (*n* = 56 participants). **c** Stronger effects of oxytocin on increasing the valuation of positive social evaluation were found in individuals who scored higher on the BDI (*n* = 56 participants). Each solid line represents the least-squares fit with shading showing the 95% CI.

We first examined the relationship between healthy individual’s depressive score and the valuation of social evaluation, as depressive individuals have been shown to have a negative bias^28,29^ and reduced learning of positive social feedback^30^. Participants’ depressive scores was measured by the Beck Depression Inventory (BDI^37^). We correlated participants’ BDI scores with unknown aversion. We found that under placebo, the BDI scores were negatively correlated with unknown aversion for positive social evaluation (indicated by *β*_*positive*_ derived from social evaluation model, *r* = −0.351, *p* = 0.008, Fig. 6a) but not for negative social evaluation (*r* = −0.15, *p* = 0.282, Fig. 6b), suggesting less unknown aversion for social evaluation (positive ones in particular) in individuals who scored higher in depressive symptoms.

Next, we examined individual differences in the effect of oxytocin on social evaluation. Interestingly, we found that the relationship between BDI scores and unknown aversion for positive social evaluation was dampened by oxytocin. Unknown aversion did not vary significantly with BDI scores after oxytocin administration (*ps* > 0.3). Furthermore, the oxytocin effect on *β*_*positive*_ (i.e., *β*_*positive-oxytocin*_ – *β*_*positive-placebo*_) was positively correlated with depression scores (*r* = 0.269, *p* = 0.045, Fig. 6c), suggesting stronger oxytocin effects on increasing aversion towards not-knowing positive social evaluation in more depressive individuals. In addition, the BDI score was not related to unknown aversion for nonsocial evaluation under placebo (*r* = −0.061, *p* = 0.710), nor did it modulate the oxytocin effect (*r* = 0.019, *p* = 0.910).

## Discussion

Knowing how the self is perceived and evaluated helps individuals to form and update self-image, appropriately interact with others, and fit in the social world^2,15,16,18,19^. The current study examined whether and how individuals value the opportunity to know opinions on the self. We demonstrate the subjective values individuals placed on knowing the evaluation of the self and further reveal how such subjective value is computed and modulated by the evaluation source (i.e., evaluation from other people or a computer program) and valence (i.e., evaluation on positive or negative aspects of the self). We show that whether the subjective value of evaluation is modulated by valence depends on the evaluation source. Specifically, the value assigned to positive and negative nonsocial evaluations is comparable and computed via a shared unknown aversion. In contrast, the value placed on positive and negative social evaluations is computed via independent unknown aversion parameters, resulting in higher value assigned to knowing positive social evaluation. Taken together, we show evidence that people care about the image of the self in others’ eyes to the extent that they would forgo monetary reward to the opportunity to know evaluations of the self.

The willingness to forgo monetary reward to the opportunity to know indicated individual’s intrinsic motive to know evaluations of the self. In the current pay-to-know choice task, both ‘to-know’ and ‘not-to-know’ options are associated with monetary rewards (or both with monetary loss in the loss condition in Exp. 3). The subjective value comparison is between ‘to-know’ and ‘not-to-know’. This is different from directly asking participants to report the amount of money that they would pay for receiving evaluation where ‘to-know’ is always associated with monetary loss, and ‘not-to-know’ is always associated with monetary saving. Thus, the pay-to-know task allowed us to dissociate ‘to-know’/‘not-to-know’ and monetary gain/loss and avoid potential influences of endowment effect and loss aversion. Despite these differences, we would expect a similar pattern of results if we asked participants to directly report their willingness to pay. As we observed similar results in the monetary gain and loss conditions, and similar results in the desirability rating that involved no monetary cost. These findings suggested that the subjective value assigned to know evaluation might be independent of specific paying methods.

The next question is what drives such a motive to know evaluations of the self. To quantify the driving force behind the costly-to-know behavior, we built a range of computational models and identified an individual-specific unknown aversion parameter (although varied widely but exhibited in most individuals), as a key determinant of the willingness to know despite monetary cost. Receiving positive evaluations is associated with self-approval or perceived as social reward^1,38,39^, whereas receiving negative evaluations could guide individual’s future behavior. Thus, being unknown may serve as a psychological cost associated with losing the opportunity of knowing the self, as being unknown about the evaluation may cause uncertainty about how others view the self and how to behave in future social interactions or to manage one’s reputation^40^. It should be noted that although it has been shown that individuals are motivated to receive positive feedback and that being praised is rewarding^1,38,39^, our finding of placing subjective value on the opportunity to know evaluation could not be simply explained by the motivation related to being praised. First, all evaluations were designed to be given after the experiment rather than immediately after participants chose the ‘to-know’ option. Second, in the current study, individuals were willing to forgo monetary reward to know evaluations of both positive and negative aspects.

Moreover, the driving force behind wanting to know social and nonsocial evaluations might be different. Individuals’ choices for the opportunity to know nonsocial evaluation were characterized by the unknown aversion computation shared by positive and negative aspects, i.e., participants’ choices of and the subjective value assigned to nonsocial evaluation were independent of the valence of the evaluation aspects. When facing nonsocial evaluation, individuals are motivated to acquire an accurate self-image by knowing how the self is objectively evaluated, as we would expect objective evaluations from a computer program. However, wanting to know social evaluation may not be driven by seeing an objective self in others’ eyes. Individuals computed the unknown aversion separately for positive and negative social evaluations and placed lower value on the negative ones. While positive social feedback conveys social approval and conformity^19,41^, negative social feedback signals social rejection^20,21^. Thus, being unknown about negative social feedback would help us avoid potential social rejection or hurt.

The evaluation of the self is an important way to be aware of how the self is perceived and to build self-image^17,30,42^. An individual’s self-image is formed and updated as a result of how the individual sees the self, as well as how others see the individual, which are respectively linked to the private and public self-image^43,44^. Public self-image, reflecting the self in the presence of others^45^, is mainly formed and updated based on social evaluations. Our finding of the positively biased valuation of social evaluation (i.e., higher subjective value and stronger unknown aversion for positive social evaluations) may provide a cognitive path to facilitate a positive public self-image. This is further supported by the result of undervalued positive social evaluation in individuals with high depressive scores, who are less motivated to manage a positive public self-image^46,47^. On the other hand, findings of equal subjective value and shared unknown aversion for positive and negative nonsocial evaluations implied the need for an objective and unbiased self-image in private^17,48^. Thus, our finding furthered our understanding of public and private self-images in that public and private self-images are distinguished by the different goals of managing a positive self-image in public and maintaining an accurate self-image in private.

Interestingly, we observed an oxytocin effect on the valuation of social (rather than nonsocial) evaluation, decreasing the subjective value and unknown aversion for negative social evaluation to the extent of having no subjective value and no unknown aversion towards negative social evaluation. This was consistent with previous animal and human studies of the attenuated processing of negative social information by oxytocin. For example, oxytocin dampened learning of negative social feedback^22^ and the influence of negative facial expressions on behaviors^24,49^. Although oxytocin did not influence the valuation of positive social evaluation in general, we observed a modulatory effect of depressive score on the oxytocin effect, showing a selective effect of oxytocin on increasing unknown aversion of positive social evaluation in individuals with higher depression scores. These findings may implicate the potential of oxytocin in alleviating social anhedonia and blunted responses to social reward, typical symptoms of depression^50–54^. This is also consistent with previous findings of a stronger oxytocin effect in less socially adapted individuals^26,27^.

It should be noted that we only recruited male participants in the current study. This choice was made to avoid potential sex differences in oxytocin effects shown in previous studies^26,55,56^, as well as to allow comparison of basic results patterns across experiments. It has been shown that females were more sensitive to positive reputation and social reward^57,58^. Moreover, females showed stronger fearful feelings for negative evaluations and social rejection than males^59^. We would speculate sex differences in the processing of evaluations. Specifically, we would expect that females (relative to males) assign higher value for the opportunity of knowing positive evaluations whereas smaller unknown aversion for negative evaluations. These possibilities need to be tested by future research.

## Methods

### Ethics approval

The experimental procedure was in line with the standards set by the Declaration of Helsinki and was approved by a local ethics committee at the State Key Laboratory of Cognitive Neuroscience and Learning, Beijing Normal University (Beijing, China). All participants provided written informed content after the experimental procedures had been fully explained and acknowledged their right to withdraw at any time during the experiment. The current study especially the hypotheses of the valuation of social evaluation and the oxytocin effects were preregistered (https://osf.io/ezxws) via the Open Science Framework with all materials and data available at https://osf.io/f6djw/.

### Participants

We recruited 375 paid males in the current study: Exp. 1: *n* = 36, age = 22.36 ± 2.75 years; Exp. 2: *n* = 36, age = 22.72 ± 2.39 years; Exp. 3: *n* = 208, age = 22.24 ± 3.49 years; Exp. 4: *n* = 56, age = 21.21 ± 2.76 years; Exp. 3: *n* = 39, age=21.82 ± 2.95 years). Participants were recruited through online advertisements and campus flyer recruitment. All participants were healthy, right-handed, free of current or a history of neurological or psychiatric disorders, and had normal or corrected-to-normal vision. Given that previous studies reported sex differences in oxytocin effects on social cognition and behavior^26,55,56^, similar to most previous oxytocin studies^23,60,61^, we only recruited male participants for the current oxytocin experiments (i.e., Exp. 4 and 5). Moreover, to allow comparison across all the experiments, as well as to avoid potential sex differences in reward-related processing shown in previous studies^57,58^, we only recruited males for Exp. 1–3. In addition, in the discovery experiments (Exp. 1 and 2) and the oxytocin experiments (Exp. 4 and 5), we only recruited single males to avoid the potential influence of romantic relationship status on social motivation^62^. In the replication experiment (Exp. 3), we recruited participants regardless of their romantic relationship status and further showed that costly-to-know behavior was not modulated by romantic relationship status (Supplementary Tables 6, 7). Participants were paid for their presence for the experiment (i.e., $10 show-up fee) plus their earnings in two randomly selected trials of the pay-to-know task (or minus that in the loss version of pay-to-know task in Exp. 3). Participants were told that, at the end of the experiment, the tokens in the pay-to-know task would be transferred into monetary incentives and the monetary gain or loss of the pay-to-know task will be added to or subtracted from their show-up fee.

### Sample size estimation

We conducted sample size estimation using G*Power 3.1.9.2^63^ to calculate the number of participants needed for the experiments in the laboratory (Exp. 1, 2, 4, and 5) to detect a reliable effect with α = 0.05 and power = 0.8. Given that no previous studies have examined the valuation of choosing to know evaluations, we based on a medium effect size of *Cohen’s d* = 0.5^64,65^ to estimate the sample size for Exp. 1 and 2 (which were conducted in parallel) and revealed that a sample of 34 participants was needed to detect a reliable effect when testing the knowing ratio against that occurring by random choice (50%) in a one-sample t-test. Exp. 1 and 2 each recruited a sample of 36 participants. In the online experiment (Exp. 3), as an online replication of our results, we recruited a large sample of 208 participants.

For the oxytocin experiment (Exp. 4), the sample size estimation was based on an estimated effect size revealed in a meta-analysis of oxytocin effects^66^ (*Cohen’s d* = 0.35). The G*Power calculation suggested that 52 participants were needed to detect a reliable interaction effect in a 2 (valence: positive vs. negative)-by-2 (treatment: oxytocin vs. placebo) within-subjects design. We thus recruited 56 participants in Exp. 4. Based on the effect size from the original finding in Exp. 4 (*Cohen’s d* = 0.41), we conducted power analysis and predetermined that 37 participants were required for a reliable effect. Thus, 39 participants were recruited in Exp. 5.

### Experimental design

We conducted five experiments (all male participants) in the current work. We first examined the subjective value individuals placed on the opportunity to know evaluations from other people (valuation of social evaluation, Exp. 1, *n* = 36) or from a computer program (valuation of nonsocial evaluation, Exp. 2, with an independent sample of *n* = 36). To reveal the similar and different valuations of social and nonsocial evaluations, we further compared data from Exp. 1 and 2, treating evaluation source (social vs. nonsocial) as a between-subjects factor. In a third experiment, we sought to replicate the findings of the valuation of social and nonsocial evaluations observed in Exp. 1 and 2 in an independent online experiment with a large sample (Exp. 3, *n* = 208, directly including the evaluation source (social vs. nonsocial) as a between-subjects factor). To examine the role of oxytocin in the valuation of social evaluation, we then employed a within-subjects, double-blind, placebo-controlled design where we compared the valuation of social evaluation under oxytocin vs. placebo administration (Exp. 4, *n* = 56). Finally, to determine whether the effect of oxytocin could also be observed on nonsocial evaluation or specific to social evaluation, we conducted Exp. 5 (*n* = 39) similarly with a within-subjects, double-blind, placebo-controlled design.

### General procedure

In Exp. 1–2, participants were invited to two separate sessions (with a mean interval of 3.86 days, Fig. 1). In the first session, participants provided self-introduction materials (Supplementary Table 8), based on which evaluation would be made. In the second session, participants completed mood-related questionnaires, the pay-to-know choice task, and a post-experiment rating survey. Responses from participants were recorded through Psychtoolbox−3.0.13 and MATLAB.

Exp. 3 was an online experiment (via Qualtrics) aiming to replicate the effects, especially the different modulation of value on the valuation of social and nonsocial evaluations that we observed in Exp. 1 and 2. Moreover, a situation where both the ‘to-know’ and ‘not-to-know’ options were associated with monetary loss was included to test whether participants were willing to lose more money for the opportunity to know evaluation. To minimize the influence of specific trait words, we used the same set of trait words for the social and nonsocial conditions. We thus employed a between-subjects design for evaluation source to avoid any potential effects of practice or facing the opportunity to know the same aspects twice in Exp. 3. Participants were randomly assigned to the social or nonsocial source.

For the oxytocin experiments (Exp. 4 and 5), we employed a double-blind, placebo-controlled, within-subjects design where participants come to the oxytocin and placebo sessions (at least 7-day interval, mean interval = 10.9 ± 6.3 days) after the first self-introduction session. The treatment order was randomized and counterbalanced across participants. There was no effect of treatment order (Exp. 4: *α*, *F*(1,54) = 2.29, *p* = 0.136; *β*_*positive*_, *F*(1,54) = 1.74, *p* = 0.193; *β*_*negative*_, *F*(1,54) = 0.06, *p* = 0.814; Exp. 5: *α*, *F*(1,37) = 0.35, *p* = 0.556; *β*, *F*(1,37) = 1.56, *p* = 0.219).

For the oxytocin and placebo sessions, participants were instructed to refrain from smoking or drinking (except water) for 2 h before the experiment. Upon arrival, participants first completed the Positive and Negative Affect Schedule^67^ to measure pre-treatment mood. Approximately 35 min before the main *pay-to-know* choice task, participants intranasally self-administered a single dose of 24 IU oxytocin or placebo (containing the active ingredients except for the neuropeptide) under an experimenter’s supervision. The procedure of oxytocin and placebo administration was similar to that used in the previous work that showed reliable effects of oxytocin on social cognition and behaviors^22,23,60^. The spray was administered to participants three times, with each administration consisting of one inhalation of 4 IU into each nostril. A 24 IU dose of oxytocin, commonly used in the oxytocin literature^23,68^, was employed in the current study because it has been recently shown to produce more pronounced effects (than 12 or 48 IU) on socio-affective processing, as well as on plasma and saliva oxytocin levels^69^.

### Pay-to-know choice task

Participants were first asked to provide one of their own photos and a self-introduction essay including their name, age, personality traits, likes/dislikes, hobbies, interests, etc. (see Supplementary Table 8 for the information that participants need to provide). We then asked participants for permission to show their self-introduction to other people or enter it into a computer program that specialized in text and facial analysis and told participants that other people or the computer program would make evaluations (rating scores for the chosen aspects) on them based on the self-introduction materials. About 4 days later (mean interval across participants: mean ± SD = 3.86 ± 4.42 days), participants were invited to the main experiment session and completed the pay-to-know choice task where participants chose between two options: ‘to-know’ (TK) and ‘not-to-know’ (NTK) the evaluations; these options differed in the amount of monetary reward received (payoff differences between ‘to-know’ and ‘not-to-know’ options, Δ*m* = *M*_*TK*_ – *M*_*NTK*_, ranged from −3 to 3).

In the pay-to-know choice task, participants completed 126 trials (63 positive traits and 63 negative traits, Supplementary Table 9) in two blocks. We designed seven payoff situations (Supplementary Table 1), with ‘to-know’ option (relative to ‘not-to-know’ option) associated with: (1) earning three more tokens, (2) earning two more tokens, (3) earning one more tokens, (4) the same amount of tokens, (5) loss of three more tokens, (6) loss of two more tokens, (7) loss of one more tokens. There were 18 trials (nine positive and nine negative traits) for each of the seven situations. The positive and negative traits were matched on arousal rating, and were randomly presented across the two blocks. Each trait word was randomly paired a monetary payoff for each participant. For each trial (Fig. 1), participants were first presented with a trait word and the evaluation source (the face of a stranger or the text-analysis program) for 4 s. Participants were then asked to choose between two options within 4 s, i.e., a ‘to-know’ option and a ‘not-to-know’ option that were associated with different amounts of tokens. For the oxytocin experiments, the same set of trait words was used. To test order effect, we included session time as a within-subjects factor. There was no difference between the first and second sessions (Exp. 4: *α*, *F*(1,55) = 0.01, *p* = 0.909; *β*_*positive*_, *F*(1,55) = 0.72, *p* = 0.399; *β*_*negative*_, *F*(1,55) = 0.12, *p* = 0.729; Exp. 5: *α*, *F*(1,37) = 2.87, *p* = 0.098; *β*, *F*(1,37) = 0.30, *p* = 0.590).

At the end of the second session of Exp. 1 and 2, we asked participants to report their subjective feelings of evaluations from another person or a computer. Participants reported their desirability (subjective desire without any cost) to know social or nonsocial evaluations. There was a significant effect of evaluation source on the desirability rating (*F*(1,67) = 14.57, *p=* 2.98 × 10^−4^, *η*^*2*^ = 0.18), suggesting a stronger desire to know social evaluation when cost was not involved. We also asked participants to report their positive (or negative) affect imagining when they received positive (or negative) evaluations. We compared the positive (or negative) affective responses between social or nonsocial evaluation. Participants showed no significant difference in negative affect rating on receiving negative evaluation between social and nonsocial source (*F*(1,67) = 0.42, *p* = 0.522). The analysis on positive affect rating showed stronger positive affect when imagining they received positive evaluations from other person than computer program (*F*(1,67) = 16.18, *p=* 1.49 × 10^−4^, *η*^*2*^ = 0.20).

In Exp. 4 and 5, at the end of each oxytocin or placebo session, participants completed a post-experiment rating of the trait words (including valence and self-relevance rating). Participants also completed the Beck Depression Inventory (BDI^37^) to access their depressive scores. The range of BDI in the current study was similar as previous studies using Asian sample^22,70,71^.

### Trait words selection

We conducted a pilot study to select trait words for the main experiment. We aimed to identify positive and negative trait words that were different in valence but matched on arousal. We recruited 33 male participants (age: 22.88 ± 3.35) and asked them to rate on the valence (from −4 to 4: extremely negative to extremely positive) and arousal (extremely calming to extremely exciting) of 307 trait words that were selected on the basis of a comprehensive list of trait adjectives^72^ and the Chinese Personality Adjective List^73^. Based on the rating scores, we included 63 positive (e.g., responsible, handsome, romantic, knowledgeable) and 63 negative words (e.g., lazy, greedy, immature, superficial) in the main experiments (Supplementary Table 9). The positive and negative trait words were significantly different in valence (*t* = 15.38, *p=* 2.22 × 10^−16^, 95% CI, 3.89–5.08, Cohen’s *d* = 2.68) but matched on arousal ratings (*t* = 1.53, *p* = 0.136, 95% CI, −0.15 to 1.08, Cohen’s *d* = 0.27).

### Data analyses

In each experiment, we asked participants to rate the self-relevance of all 126 trait words (−10 = not like me at all, 10 = exactly like me). On the basis of the self-relevance rating, we divided the trait words into self-relevant and self-irrelevant traits for each participant using medium split on self-relevance rating. To prevent spurious confounds of self-relevance on the valuation process^3^, formal data analysis was performed on the self-relevant trait words (the trait words of which the self-relevance rating were higher than the mean rating scores of all trait words) on a participant basis and excluded the self-irrelevant traits (the trait words of which the self-relevance rating were lower than the mean rating scores of all trait words). In addition, evaluations on the positive and negative aspects (with high self-relevance) are most likely to reflect positive and negative evaluations on the self.

For the model-free indices, we mainly focused on the overall knowing ratio and costly knowing ratio. All the indices were calculated separately for trials of positive and negative traits. (1) The overall knowing ratio was the proportion of choosing the ‘to-know’ option in all trials; (2) the costly knowing ratio was the proportion of choosing the ‘to-know’ option in costly-to-know trials where choosing the ‘to-know’ option gains no more money than choosing the ‘not-to-know’ option.

To quantify the subjective value participants placed on knowing, each participant’s choice data was fit with sigmoid functions to give an estimation of the point of subjective equivalence (PSE) between the ‘to-know’ and ‘not-to-know’ options.

The sigmoid function is defined as follows:

1 $$f\left( x \right) = m_i + \frac{{m_j - m_i}}{{1 + 10^{ - \beta \left( {x - \alpha } \right)}}}$$

2 $${\mathrm{PSE}} = f^{\prime} \left( {0.5} \right)$$

where *x* represents the payoff differences between ‘to-know’ and ‘not-to-know’ options (i.e., ∆*M=M*_*TK*_ – *M*_*NTK*_), *f(x)* represents the knowing ratio in each payoff difference condition, *m*_*i*_ represents the lower asymptote, *m*_*j*_ represents the upper asymptote, *β* represents the slope of the sigmoidal response function, and *α* represents the x-value of the sigmoid midpoint. The PSE was calculated as the monetary value at which a participant effectively chose arbitrarily between the ‘to-know’ and ‘not-to-know’ options (Eq. (2)). The estimated PSEs were obtained through a bootstrap procedure with 200 iterations, similar to the previous work^31^. Sigmoid curves were fit to the knowing ratio for each bootstrap sample by implementing nonlinear least-squares estimates in R. The goodness of fit of each bootstrap sample to the sigmoid function was measured, and the sigmoid function fitted well with each sample (r^2^ ranging from 0.932 to 1.000 across all experiments).

### Computational modeling

We fit a range of models to each participant’s choice data to capture the valuation process of choosing to know evaluation on a trial-by-trial basis. For each model, trial-by-trial value differences (i.e., ∆*V*) were transformed into choice probabilities using a softmax function:^74,75^

3 $$P\left( {{\mathrm{choose}}\;{\mathrm{the}}\;{\mathrm{left}}\;{\mathrm{choice}}} \right) = \left( {\frac{1}{{1 + {\mathrm{e}}^{ - \gamma \Delta V}}}} \right)\left( {1 - 2\varepsilon } \right) + \varepsilon$$

where γ is an inverse temperature parameter capturing the sensitivity of choices to Δ*V* and *ε* is a lapse rate that captures choice noisiness resulting from factors independent of Δ*V*. These parameters were estimated for each participant by implementing nonlinear optimization in MATLAB (MathWorks, Inc.).

A Bayesian model comparison technique^76,77^ was employed to compare models. For each model, we computed the Bayesian Information Criterion (BIC) scores for each participant. We then compared the group BIC scores (summed BIC scores across participants) and identified the best-fit model with the lowest group BIC score. We showed that Model 1 (2), for social (nonsocial) evaluation choices, outperformed a range of alternative models, including those considering temporal discounting across trials or monetary loss aversion (Supplementary Table 2).

### Statistics and reproducibility

The oxytocin experiments were double-blind; that is, both participants and experimenters were blind to the treatment administration. Data analysis was not performed blind to the conditions of the experiments. We first conducted one-sample *t*-test to compare overall knowing ratio with 50%. To examine the valence effect on valuation of evaluation and how evaluation source modulated the valence effect, we performed 2-by-2 ANOVAs with valence (positive vs. negative) as a within-subject factor and evaluation source (social vs. nonsocial) as a between-subject factor. Significance test for PSE was performed by calculating bootstrap confidence interval based on 200 bootstrap replicates. To evaluation the oxytocin effect on valuation, we conducted paired *t*-test and Chi-square test. Data distribution was assumed to be normal but this was not formally tested. All correlations were performed by Person’s correlation coefficient analysis. Results were considered significant at *p* < 0.05. Results from bootstrap analysis were considered significant when zero was not included in bootstrap confidence interval. Cohen’s *d* for dependent samples indicate the standardized mean change as calculated by dividing the mean difference scores by the standard deviation of these difference scores.

### Reporting summary

Further information on research design is available in the Nature Research Reporting Summary linked to this article.

## Supplementary information

Supplementary Information

Reporting Summary

## Data Availability

The data that support the findings of this study are available at https://osf.io/f6djw/.

## Code availability

Analysis code to model the unknown aversion towards social and nonsocial evaluation is available at https://osf.io/f6djw/.
