# Supplementary material for: Oxytocin facilitates valence-dependent valuation of social evaluation of the self
Source: Commun Biol. 2020 Aug 13;3:433. doi: 10.1038/s42003-020-01168-w (PMC7426917; doi:10.1038/s42003-020-01168-w)
Supplement: Supplementary file 1 — Supplementary Information [file 42003_2020_1168_MOESM1_ESM.pdf]

Supplementary Tables

Supplementary Table 1. Payoff matrix for the pay-to-know choice task.

	Reward condition (for all experiments)			Loss condition (for the loss version in Exp. 3)		
Payoff difference "TK − NTK"	To-know Payoff	Not-to-know Payoff	No. of Trials	To-know Payoff	Not-to-know Payoff	No. of Trials
+3	+4	+1	18	-1	-4	18
+2	+4	+2	9	-1	-3	9
	+3	+1	9	-2	-4	9
+1	+4	+3	6	-1	-2	6
	+3	+2	6	-2	-3	6
	+2	+1	6	-3	-4	6
0	+4	+4	4	-4	-4	4
	+3	+3	5	-3	-3	5
	+2	+2	5	-2	-2	5
	+1	+1	4	-1	-1	4
-1	+1	+2	6	-4	-3	6
	+2	+3	6	-3	-2	6
	+3	+4	6	-2	-1	6
-2	+1	+3	9	-4	-2	9
	+2	+4	9	-3	-1	9
-3	+1	+4	18	-4	-1	18

Supplementary Table 2. Bayesian Information Criterion (BIC) for all models.

Models	Parameters	No. of Parameters per participant	Model BIC Exp1	Model BIC Exp2	Model BIC Exp3 social	Model BIC Exp3-nonsocial	Model BIC Exp4 PL	Model BIC Exp5 PL
Model 1 (M1)	α, $\beta_{positive}$, $\beta_{negative}$	5	**2173.03**	1967.87	**6974.80**	7985.59	**3367.77**	2147.13
Model 2 (M2)	α, β	4	2205.95	**1944.32**	7031.25	**7979.17**	3439.63	**2144.55**
Model 3 (M3)	κ	3	3689.24	3164.42	9508.37	10122.87	5564.71	3524.01
Model 4 (M4)	$\kappa_{positive}$, $\kappa_{negative}$	4	3724.52	3224.94	9633.11	10249.43	5588.09	3580.82
Model 5 (M5)	$\alpha_{positive}$, $\alpha_{negative}$, β	5	2241.54	1986.44	7123.71	8090.72	3483.68	2193.48
Model 6 (M6)	$\alpha_{positive}$, $\alpha_{negative}$, $\beta_{positive}$, $\beta_{negative}$	6	2234.15	2028.86	7118.95	8166.79	3468.92	2214.99
Model 7 (M7)	κ, $\delta_{positive}$, $\delta_{negative}$	5	2918.96	2579.80	NaN[a]	NaN[a]	4321.65	2800.74
Model 8 (M8)	κ, $\delta_{positive}$, $\delta_{negative}$	5	2909.65	2737.54	NaN[a]	NaN[a]	4363.82	3053.44
Model 9 (M9)	α, $\beta_{positive}$, $\beta_{negative}$, λ_m	6	2249.56	2049.69	7146.16	8145.09	3493.08	2235.12
Model 10 (M10)	α, β, $\lambda_{m\text{-}positive}$, $\lambda_{m\text{-}negative}$	6	2281.62	2112.86	7224.42	8220.10	3595.47	2258.58

Note:

Model 1 (2) fitted best participants' choices in the social (non-social) pay-to-know task in a model comparison that considers differences in model complexity. More complex model variants included separate parameters for the positive-trait and negative-trait conditions, discount rate for outcome, and loss aversion for monetary payoff. The Bayesian Information Criterion (BIC) scores are the Bayesian equivalent to a fixed effects analysis.

[a]It should be noted that the choice data of the online experiment (i.e. Exp.3) was not fitted with models which considered temporal discounting process (i.e. M7 and M8) as the trial sequence was not recorded by Qualtrics platform.

Supplementary Table 3. Pre-experiment and post-experiment mood, mood change, and post-experiment rating scores on attitude under oxytocin and placebo administration in Exp. 4

	**Oxytocin**		**Placebo**		**Oxytocin vs. Placebo**			
	**mean**	**std**	**mean**	**std**	***t***	***p***	**95% *CI***	**Cohen's *d***
**Mood**								
Pre-positive	29.12	7.24	27.96	7.23	1.33	0.188	-0.58, 2.88	0.18
Pre-negative	14.75	4.55	15.14	6.42	-0.45	0.653	-2.13, 1.35	-0.06
Post-positive	27.84	8.38	26.45	8.95	1.31	0.195	-0.73, 3.52	0.18
Post-negative	14.23	4.61	14.14	4.63	0.14	0.887	-1.16, 1.34	0.02
Δ positive	-1.28	5.75	-1.52	5.16	0.22	0.825	-1.93, 2.41	0.03
Δ negative	-0.52	3.20	-1.00	5.18	0.62	0.540	-1.08, 2.05	0.08
**Post-rating**								
Influence of monetary payoff	5.7	2.55	6.11	2.52	-1.55	0.128	-0.12, 0.95	0.21

Note:
Δ positive = Post-positive – Pre-positive; Δ negative = Post-negative– Pre-negative.
Participants receiving oxytocin and placebo did not differ in mood before and after the treatment. Moreover, participant's mood change before and after the treatment and post-rating were not influenced by receiving oxytocin and placebo.

Supplementary Table 4. Pre-experiment and post-experiment mood, mood change, and post-experiment rating scores under oxytocin and placebo administration in Exp. 5

	**Oxytocin**		**Placebo**		**Oxytocin vs. Placebo**			
	**mean**	**std**	**mean**	**std**	***t***	***p***	**95% *CI***	**Cohen's *d***
**Mood**								
Pre-positive	31.61	6.18	33.11	5.50	-1.72	0.093	-3.26,0.26	-0.28
Pre-negative	14.66	5.26	14.53	5.76	0.18	0.862	-1.39,1.66	0.03
Post-positive	28.56	7.88	30.69	7.30	-1.93	0.062	-4.40,0.12	-0.32
Post-negative	15.89	5.59	15.11	6.30	0.95	0.351	-0.89,2.45	0.16
Δ positive	3.28	5.64	2.53	5.20	0.76	0.451	-1.25,2.75	0.13
Δ negative	-1.72	4.15	-1.31	4.57	-0.38	0.705	-2.63,1.80	-0.06
**Post-rating**								
Influence of monetary payoff	6.15	2.39	6.15	2.19	<0.01	1	-0.52,0.52	0.00

Note:
Δ positive = Post-positive – Pre-positive; Δ negative = Post-negative– Pre-negative. Participants receiving oxytocin and placebo did not differ in mood before and after the treatment. Moreover, participant's mood change before and after the treatment and post-rating were not influenced by receiving oxytocin and placebo.

Supplementary Table 5. Mean (Std) reaction times (RTs, ms) under oxytocin and placebo administration in Exp. 4 and 5

	**Groups**	**All trials**	**Positive trait words**	**Negative trait words**
**Exp. 4**	Placebo	1120.22 (341.43)	1119.69 (341.34)	1120.76 (352.36)
	Oxytocin	1110.81 (337.15)	1109.30 (322.35)	1112.33 (632.18)
	Placebo vs. Oxytocin: *t* (*p*)	0.26 (0.796)	0.29 (0.770)	0.21 (0.837)
	Placebo vs. Oxytocin: 95% *CI*	[-0.06,0.08]	[-0.06,0.08]	[-0.07,0.09]
	Placebo vs. Oxytocin: Cohen's *d*	0.03	0.04	0.03
**Exp. 5**	Placebo	1354.51 (500.28)	1349.90 (474.49)	1359.13 (541.08)
	Oxytocin	1370.82 (396.26)	1360.73 (392.63)	1380.91 (442.66)
	Placebo vs. Oxytocin: *t* (*p*)	-0.31 (0.757)	-0.19 (0.848)	-0.37 (0.714)
	Placebo vs. Oxytocin: 95% *CI*	[-0.12,0.09]	[-0.12,0.10]	[-0.14,0.10]
	Placebo vs. Oxytocin: Cohen's *d*	-0.05	-0.03	-0.06

Supplementary Table 6. Modulation of romantic relationship status on decision-making for social evaluation in Exp.3

Variables	*F*	*p*	η^2
Knowing ratio (evaluation on positive aspects)	0.75	0.526	0.02
Costly knowing ratio (evaluation on positive aspects)	1.38	0.253	0.04
Knowing ratio (evaluation on negative aspects)	0.48	0.696	0.02
Costly knowing ratio (evaluation on negative aspects)	1.19	0.317	0.04
Model-based indices: α	2.85	0.041	0.08
Model-based indices: $\beta_{positive}$	0.19	0.900	<0.01
Model-based indices: $\beta_{negative}$	1.69	0.175	0.05

Note: The *F* and *p* values were from one-way ANOVA

Supplementary Table 7. Modulation of romantic relationship status on decision-making for non-social evaluation in Exp.3

Variables	F	p	η^2
Overall knowing ratio (evaluation on positive aspects)	0.94	0.424	0.03
Costly knowing ratio (evaluation on positive aspects)	0.44	0.727	0.01
Overall knowing ratio (evaluation on negative aspects)	0.12	0.950	<0.01
Costly knowing ratio (evaluation on negative aspects)	0.79	0.504	0.02
Model-based indices: α	0.50	0.681	0.01
Model-based indices: β	0.69	0.562	0.02

Note: The F and p values were from one-way ANOVA

Supplementary Table 8. Information that participants need to provide for the self-introduction.

	Items
Basic information	Name (or Nickname)
	Age
	Birth place
	Name of your university
	Major
Info related to personality	Please provide daily-life examples to introduce your personality (e.g. extrovert/introvert, conventional/radical, etc.).
	Pros (examples in your daily life to show your pros)
	Cons (examples in your daily life to show your cons)
Info related to likes/dislikes	Hobbies
	Favorite dressing style
	Least favorite dressing style
	Favorite book
	Favorite movie
	Your idol
	Favorite things you'd you like to do during your leisure time
Personal experiences	Most exciting moment in college
	Most ashamed moment in college
Info related to personal value	What would you prefer? Please write down your decision and reasons.<br>In major events, such as birthday party or graduation party, would you like to hold a big party and invite all the people you know? or would you like to hold a small party and just invite family members and best friends?
	How would you rank the importance of these people: Family members, friends, and girlfriend/boyfriend?

Supplementary Table 9. All trait words and rating scores of valence and arousal for each trait word.

Positive trait	Valence	Arousal	Negative trait	Valence	Arousal
knowledgeable	3.24	5.61	nasty	-2.24	5.36
mature	2.36	5.36	stingy	-1.79	6.21
outstanding	2.52	6.00	decadent	-2.30	5.24
clever	2.88	5.58	stupid	-2.21	4.21
decent	2.97	4.67	foolish	-2.24	4.91
interesting	2.70	5.94	pompous	-1.52	5.21
responsible	3.09	5.24	pessimistic	-2.42	4.94
neat	2.67	5.24	unnatural	-1.73	4.45
gregarious	2.30	5.21	sloppy	-1.58	4.79
kindly	1.67	4.55	superficial	-2.03	4.48
honest	2.97	5.00	vindictive	-2.12	4.88
active	2.76	4.88	old-fashioned	-1.76	4.61
witty	2.39	5.36	reckless	-0.88	4.48
prominent	3.09	5.97	outrageous	-1.18	5.03
dutiful	2.94	5.64	shallow	-0.85	4.76
motivated	2.76	5.33	irritable	-1.82	5.12
energetic	2.79	5.36	rude	-1.85	4.45
dedicated	2.21	5.21	suspicious	-1.09	5.18
outgoing	3.15	5.48	brash	-1.27	4.79
lovely	2.64	5.97	ugly	-0.27	4.64
reliable	2.97	6.30	weak	-0.52	4.18
trusted	2.76	6.15	rigid	-1.42	5.39
romantic	2.39	5.45	picky	-3.21	5.58
optimistic	2.27	6.24	narrow	-2.42	4.94
dispassionate	3.09	5.73	extreme	-2.82	5.24
powerful	2.30	4.97	feeble	-2.24	4.42
flexible	2.52	5.09	vulgar	-2.00	4.97
attractive	2.27	6.24	impulsive	-2.82	5.24
charming	2.12	6.21	careless	-1.42	5.27
capable	2.85	5.55	boring	-2.03	4.97
strong	2.61	5.39	arrogant	-2.18	5.67
enthusiastic	2.85	5.76	impatient	-2.55	4.64
zealous	2.94	5.85	indifferent	-2.06	5.36
serious	3.27	5.18	blind	-2.00	5.06
big-heart	2.18	5.79	indiscreet	-1.79	4.76
faithful	2.94	4.94	arbitrary	-2.33	5.39
handsome	2.58	5.82	heavy-headed	-2.30	4.94
jovial	2.58	5.03	negligent	-1.82	5.33

frank	2.61	5.30	conceited	-2.21	5.39
easy-going	2.42	4.73	lazy	-1.67	5.52
perfect	3.15	5.91	dull	-1.97	4.94
stable	2.73	5.61	slack	-2.15	5.33
selfless	3.00	5.06	self-abased	-1.97	4.45
practical	2.52	4.64	cursory	-1.52	5.82
careful	2.82	5.45	timid	-2.45	5.52
unrestrained	2.52	5.79	autistic	-1.64	4.67
filial	3.18	5.64	slothful	-1.79	4.73
sunny	2.82	5.36	childish	-1.39	6.15
smart	2.67	5.33	coward	-1.97	5.21
excellent	3.39	6.55	inflexible	-1.70	4.42
humorous	2.45	5.52	eccentric	-2.00	5.39
friendly	2.73	4.76	vulnerable	-1.06	4.21
talented	2.76	5.30	vacuous	-1.85	4.06
cool	2.39	5.00	loose	-1.55	4.64
righteous	3.00	5.27	fickle	0.03	5.24
sincere	2.88	5.42	idle	-1.67	4.24
moral	2.67	4.94	flamboyant	-2.33	4.52
upright	3.09	5.18	immature	-1.61	5.82
straight	2.48	4.79	shy	-2.21	5.52
single-minded	2.30	5.42	constrained	-2.15	4.79
independent	2.64	5.36	verbose	-0.55	4.24
confident	2.12	5.76	mediocre	-1.88	5.70
autonomous	2.64	5.82	vain	-0.58	4.30

42

Supplementary Figures

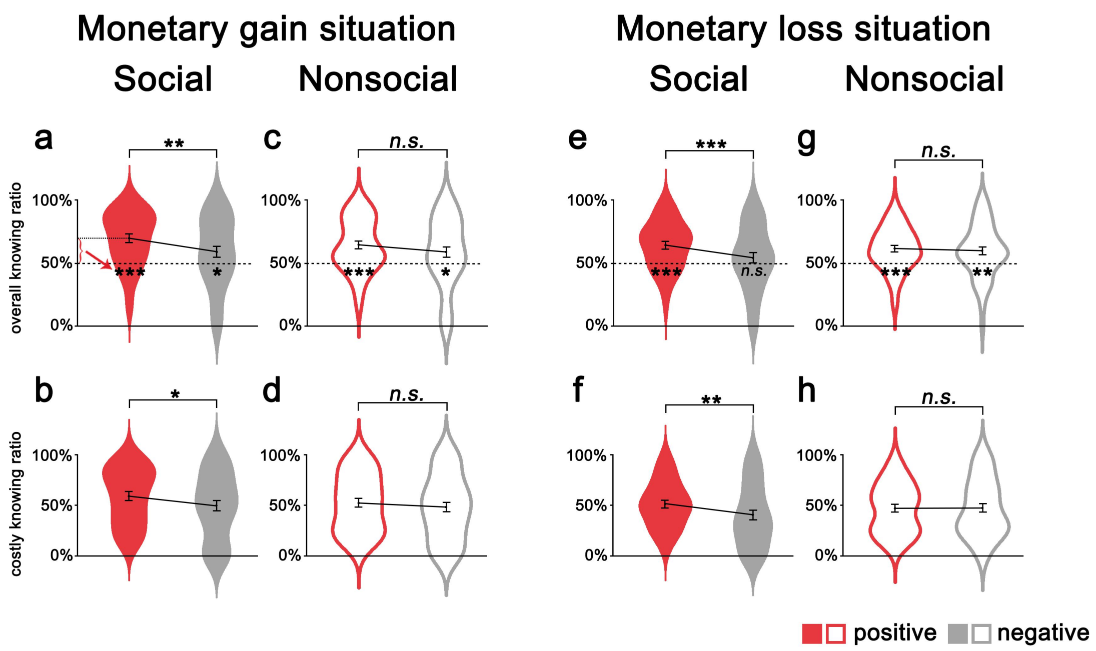

Supplementary Figure 1. *Model-free results for monetary gain and loss situations in Exp. 3.* Participants preferred to pay more to know social evaluations of positive aspects than negative aspects in both monetary gain (***a-b***) and loss situations (***e-f***), whereas they would forgo a similar amount of money for the opportunity to know positive and negative non-social evaluations, also in monetary gain (***c-d***) and loss situations (***g-h***). The violin plots indicate the distribution of indices from the pay-to-know task, with elements inside the violin plots representing the mean and standard error. (*$p < 0.05$, **$p < 0.01$ and ***$p < 0.001$; *n.s.*, not significant)

Supplementary Methods

List of alternative models for model comparison.

To arbitrate the computational processes employed by the participants, we compared a range of models, each of which explained choices in terms of the value difference (ΔV) between the left and right choices. Models 1 through 6 differed in model complexity, mainly capturing the contribution of monetary payoff difference and the contribution of knowing the evaluation on action choice. More complex model variants included independent contributions of monetary payoff differences and to-know evaluation, and separate parameters for the positive and negative trials. In Models 7 and 8, we assumed that the participant's choices changed over the course of the session and considered a parameter that captured temporal discounting of the subjective value difference between the 'to-know' and 'not-to-know' options. In Models 9 and 10, we conceptualized a loss aversion towards monetary reward, assuming that participants require more money for choosing not-to-know than they are willing to pay to know.

$$\Delta V = \alpha \Delta m + \beta \Delta e$$

$$\beta = \begin{cases} \beta_{positive} \text{ evaluation on positive aspect} \\ \beta_{negative} \text{ evaluation on negative aspect} \end{cases}$$

<u>Model 1</u>

In Model 1, the likelihood of choosing the left choice is a function of the value difference (ΔV) between the two choices. The value difference depends on the difference in monetary payoff ($\Delta m = M_{left} - M_{right}$) and to-know evaluation or not (Δe = 1, if left choice is 'to know'; Δe = -1, if left choice is 'not to know'), contribution of monetary reward (α), and unknown aversion parameter that captures the subjective cost of not-knowing evaluation. When unknown aversion approaches 1, participants are maximally averse to not-knowing; as unknown aversion approaches -1, participants are maximally averse to knowing evaluation. Moreover, we assumed that participants made decisions by separately evaluating the costs of not-knowing evaluations for positive and negative aspects, considering independent unknown aversion parameters

for positive and negative aspects (i.e. $\beta_{positive}$ and $\beta_{negative}$).

$$\Delta V = \alpha \Delta m + \beta \Delta e$$

<u>Model 2</u>

Model 2 is similar to Model 1, testing whether participants make decisions based on separate evaluation of the contribution of the monetary payoff differences and to-know evaluations or not to action choice. This model further tested whether participants considered unknown aversion to a similar degree.

$$\Delta V = -\kappa \Delta m + \kappa \Delta e$$

<u>Model 3</u>

Model 3 is only characterized by an unknown aversion, that captures the the subjective cost of not-knowing evaluation

$$\Delta V = -\kappa \Delta m + \kappa \Delta e$$

$$\kappa = \begin{cases} \kappa_{positive} \text{ evaluation on positive aspect} \\ \kappa_{negative} \text{ evaluation on negative aspect} \end{cases}$$

<u>Model 4</u>

Model 4 is similar to Model 1. In Model 2, we assumed that participants considered independent unknown aversion parameters for positive and negative aspects (i.e., $\kappa_{positive}$ and $\kappa_{negative}$, respectively).

$$\Delta V = \alpha \Delta m + \beta \Delta e$$

$$\alpha = \begin{cases} \alpha_{positive} \text{ evaluation on positive aspect} \\ \alpha_{negative} \text{ evaluation on negative aspect} \end{cases}$$

<u>Model 5</u>

Model 5 was similar to Model 2 in that it allowed for the separated contribution of monetary payoff difference and knowing evaluations but further tested whether participants considered the contribution of monetary payoff to different degrees when choosing for positive and negative aspects.

$$\Delta V = \alpha \Delta m + \beta \Delta e$$

$$\alpha = \begin{cases} \alpha_{positive} \text{ evaluation on positive aspect} \\ \alpha_{negative} \text{ evaluation on negative aspect} \end{cases}$$

$$\beta = \begin{cases} \beta_{positive} \text{ evaluation on positive aspect} \\ \beta_{negative} \text{ evaluation on negative aspect} \end{cases}$$

<u>Model 6</u>

Model 6 was similar to Model 5 but further tested whether participants considered different degrees of the contribution of monetary payoff differences on action choice for positive and negative trials.

$$\Delta V = -\kappa \Delta m + \kappa \Delta E$$

$$\Delta E = \begin{cases} \dfrac{\Delta e_t}{1 + \delta_{positive}(t - n)} \text{ evaluation on positive aspect} \\ \dfrac{\Delta e_t}{1 + \delta_{negative}(t - n)} \text{ evaluation on negative aspect} \end{cases}$$

<u>Model 7</u>

Model 7 tested the possibility that subjective value differences between the ‘to-know’ and ‘not-to-know’ options would be discounted over the course of the session by considering the temporal discounting of the subjective value difference. It is possible that participant’s motivation for choosing to know evaluation is decreased due to the fatigue effect. In model 7, Δe_t on trial t was hyperbolically discounted at a discount rate δ; n is the total number of trials. We also tested whether subjective value differences between to-know and not-to-know positive and negative evaluations would be independently discounted by considering independent discount rates for positive and negative evaluations.

$$\Delta V = -\kappa \Delta m + \kappa \Delta E$$

$$\Delta E = \begin{cases} \dfrac{\Delta e_t}{1 + \delta_{positive}(Cum_{knowing})} & \text{evaluation on positive aspect} \\ \dfrac{\Delta e_t}{1 + \delta_{negative}(Cum_{knowing})} & \text{evaluation on negative aspect} \end{cases}$$

<u>Model 8</u>

Model 8 was similar to Model 7, but this model assumed that the change in a participant's preference would be dependent upon whether more 'to-know' choices were made rather than more 'not-to-know' choices were made, as in Model 7. It is possible that the participant's preference towards knowing evaluations is weakened over the course of the session because the satisfaction increased after making enough 'to-know' choices. In model 8, Δe_t on trial t was hyperbolically discounted at a discount rate that was independent of positive and negative aspects, $\delta_{positive}$ and $\delta_{negative}$; $Cum_{knowing}$ represented the accumulative frequency of 'to-know' choices.

$$\Delta V = \alpha L_m \Delta m + \beta \Delta e$$

$$\beta = \begin{cases} \beta_{positive} & \text{evaluation on positive aspect} \\ \beta_{negative} & \text{evaluation on negative aspect} \end{cases}$$

$$L_m = \begin{cases} 1 \text{ if } \Delta m \geq 0 \\ \lambda \text{ if } \Delta m < 0 \end{cases}$$

<u>Model 9</u>

Model 9 was similar to Model 1 but further tested whether participants were loss-averse for monetary payoff (λ). Note that loss aversion, in the context of the current experiment, produces a pattern of choices in which participants require more money to forgo knowing evaluations than they are willing to pay to know when the 'to know' option is associated with larger monetary payoff, and participants require more money to choose knowing evaluations than they are willing to pay for not-to-know when the 'to-know' option is associated with smaller monetary payoff.

$$\Delta V = \alpha L_m \Delta m + \beta \Delta e$$

$$L_m = \begin{cases} 1 \text{ if } \Delta m \geq 0 \\ \lambda \text{ if } \Delta m < 0 \end{cases}$$

$$\lambda = \begin{cases} \lambda_{positive} \text{ evaluation on positive aspect} \\ \lambda_{negative} \text{ evaluation on negative aspect} \end{cases}$$

<u>Model 10</u>

Model 10 is similar to Model 2 but further tested whether participants showed different degrees of loss aversion for monetary payoff in positive and negative trials.
