# Supplementary material for: Oxytocin facilitates valence-dependent valuation of social evaluation of the self
Source: Commun Biol. 2020 Aug 13;3:433. doi: 10.1038/s42003-020-01168-w (PMC7426917; doi:10.1038/s42003-020-01168-w)
Supplement: Supplementary file 2 — Reporting Summary [file 42003_2020_1168_MOESM2_ESM.pdf]

nature research

Corresponding author(s): Yina Ma

Last updated by author(s): Jun 30, 2020

Reporting Summary

Nature Research wishes to improve the reproducibility of the work that we publish. This form provides structure for consistency and transparency in reporting. For further information on Nature Research policies, see our Editorial Policies and the Editorial Policy Checklist.

Statistics

For all statistical analyses, confirm that the following items are present in the figure legend, table legend, main text, or Methods section.

n/a	Confirmed	
☐	☒	The exact sample size (n) for each experimental group/condition, given as a discrete number and unit of measurement
☐	☒	A statement on whether measurements were taken from distinct samples or whether the same sample was measured repeatedly
☐	☒	The statistical test(s) used AND whether they are one- or two-sided<br>*Only common tests should be described solely by name; describe more complex techniques in the Methods section.*
☐	☒	A description of all covariates tested
☒	☐	A description of any assumptions or corrections, such as tests of normality and adjustment for multiple comparisons
☐	☒	A full description of the statistical parameters including central tendency (e.g. means) or other basic estimates (e.g. regression coefficient) AND variation (e.g. standard deviation) or associated estimates of uncertainty (e.g. confidence intervals)
☐	☒	For null hypothesis testing, the test statistic (e.g. F, t, r) with confidence intervals, effect sizes, degrees of freedom and P value noted<br>*Give P values as exact values whenever suitable.*
☒	☐	For Bayesian analysis, information on the choice of priors and Markov chain Monte Carlo settings
☒	☐	For hierarchical and complex designs, identification of the appropriate level for tests and full reporting of outcomes
☐	☒	Estimates of effect sizes (e.g. Cohen's d, Pearson's r), indicating how they were calculated

Our web collection on statistics for biologists contains articles on many of the points above.

Software and code

Policy information about availability of computer code

Data collection	Responses from participants were recorded through Psychtoolbox-3.0.13 and MATLAB. Online data was collected through Qualtrics platform
Data analysis	Analyses were conducted in MATAB and R. Bootstrap analysis was performed in boot toolbox in R.

For manuscripts utilizing custom algorithms or software that are central to the research but not yet described in published literature, software must be made available to editors and reviewers. We strongly encourage code deposition in a community repository (e.g. GitHub). See the Nature Research guidelines for submitting code & software for further information.

Data

Policy information about availability of data

All manuscripts must include a data availability statement. This statement should provide the following information, where applicable:

- Accession codes, unique identifiers, or web links for publicly available datasets
- A list of figures that have associated raw data
- A description of any restrictions on data availability

We declare that the data supporting the findings of this study are available within the article and its Supplementary Information files.

Field-specific reporting

Behavioural & social sciences study design

All studies must disclose on these points even when the disclosure is negative.

Study description	Quantitative experimental study.
Research sample	A total of 375 male participants took part in this study (Mean ± SD age = 22.10 ± 3.19 years). Thirty-six participants took part in Experiment 1 (Mean ± SD age = 22.36 ± 2.75 years); 36 participants in Experiment 2 (Mean ± SD age = 22.72 ± 2.39 years); 208 participants in Experiment 3 (Mean ± SD age = 22.24 ± 3.49 years); 56 participants in Experiment 4 (Mean ± SD age = 21.21 ± 2.76 years); 39 participants in Experiment 5 (Mean ± SD age = 21.82 ± 2.95 years). Participants in Experiment 3 participated through Qualtrics platform.
Sampling strategy	We first determined minimum sample size by using G*Power 3.1.9.2. We calculated the number of participants needed for the experiments in the laboratory to detect a reliable effect with α = 0.05, power = 0.8, using the effect size from previous studies (for Exp. 1, 2, and 4) or the effect size from original finding in Exp. 4 (for Exp. 5). In Exp. 3, upon connecting to the experimental web page, participants were randomly assigned for one of the four different conditions (i.e. monetary gain/monetary loss × social/non-social). Sample size for each condition were: N = 54 (Monetary gain and non-social condition), N = 56 (Monetary loss and non-social condition), N = 48 (Monetary gain and social condition), and N = 50 (Monetary loss and social condition).
Data collection	For experiments in the laboratory (Exp. 1, 2, 4, and 5), participants pressed a key in a keyboard and their response were recorded through Psychtoolbox-3.0.13 in MATLAB. In Exp. 3, data were collected through a computer-based online task that can be played in a web browser. To minimize the risk of multiple accesses from the same person, we introduced the restriction that a single Wechat ID associated with each participant could participate only once in the experiment.
Timing	Data collection was completed between October 2017 and July 2018.
Data exclusions	No participant's data was excluded. Trials presenting self-irrelevant word were excluded in formal analyses.
Non-participation	One participant in Exp. 1 and two participants in Exp. 4 dropped out before finishing the main task.
Randomization	Participants in Exp. 3 were randomly allocated to one of the four conditions. Both Exp. 4 and 5 used a within-subject design and participants came twice and were assigned to oxytocin or placebo administration for each time. The sequence of oxytocin/placebo administration was randomly determined.

Reporting for specific materials, systems and methods

We require information from authors about some types of materials, experimental systems and methods used in many studies. Here, indicate whether each material, system or method listed is relevant to your study. If you are not sure if a list item applies to your research, read the appropriate section before selecting a response.

Materials & experimental systems

n/a	Involved in the study	
☒	☐	Antibodies
☒	☐	Eukaryotic cell lines
☒	☐	Palaeontology and archaeology
☒	☐	Animals and other organisms
☐	☒	Human research participants
☒	☐	Clinical data
☒	☐	Dual use research of concern

Methods

n/a	Involved in the study	
☒	☐	ChIP-seq
☒	☐	Flow cytometry
☒	☐	MRI-based neuroimaging

Human research participants

Policy information about studies involving human research participants

Population characteristics	375 males, mean age was 22.10 ± 3.19 years.
Recruitment	All participants were university undergraduate and graduate students and were recruited through online advertisements and campus flyer recruitment. They were paid for their participation and were unaware of the purpose of the study.
Ethics oversight	Local ethics committee at the State Key Laboratory of Cognitive Neuroscience and Learning, Beijing Normal University (Beijing, China)

Note that full information on the approval of the study protocol must also be provided in the manuscript.
